# The role of cyclic di-GMP in biomaterial-associated infections caused by commensal *Escherichia coli*

**DOI:** 10.1371/journal.pone.0330229

**Published:** 2025-08-20

**Authors:** Shanshan Li, Chengwei Bi, Bingquan Xiang, Zhonghui Wang, Hui Yang, Chaojiang Fu, Lianpu Chen, Ying Chen

**Affiliations:** 1 Department of Anesthesiology, The Third Affiliated Hospital of Kunming Medical University, Yunnan Cancer Hospital, Peking University Cancer Hospital Yunnan, Kunming, China; 2 Department of Thoracic Surgery I, The Third Affiliated Hospital of Kunming Medical University, Yunnan Cancer Hospital, Peking University Cancer Hospital Yunnan, Kunming, China; 3 Department of Urinary Surgery, The Third Affiliated Hospital of Kunming Medical University, Yunnan Cancer Hospital, Peking University Cancer Hospital Yunnan, Kunming, China; 4 Intensity Care Unit, The Third Affiliated Hospital of Kunming Medical University, Yunnan Cancer Hospital, Peking University Cancer Hospital Yunnan, Kunming, China; 5 Department of Emergency Medicine, The Third Affiliated Hospital of Kunming Medical University, Yunnan Cancer Hospital, Peking University Cancer Hospital Yunnan, Kunming, China; University of Pennsylvania, UNITED STATES OF AMERICA

## Abstract

Biofilms are protective structures that bacteria use to evade the immune system and resist antibiotics, leading to complications in medical treatments, especially with implanted devices. The molecule cyclic di-GMP (c-di-GMP) is crucial for biofilm formation in *Escherichia coli* (*E. coli*). To understand its role in biomaterial-associated infections (BAIs), we created four *E. coli* strains with varying c-di-GMP levels: a knockout strain (Δ*dgcQ*), an overexpression strain (O*dgcQ*), a complemented strain (CΔ*dgcQ*), and a wild-type mutant strain (WT). By employing *in vitro* BAI models and techniques such as crystal violet (CV) staining, XTT assay, confocal laser scanning microscopy (CLSM), and scanning electron microscopy (SEM), we observed that the Δ*dgcQ* strain, with low c-di-GMP levels, adhered more readily to biomaterial surfaces at the initial stage of biofilm formation, yet faced difficulties in sustaining mature biofilms. In contrast, O*dgcQ* and CΔ*dgcQ* with higher c-di-GMP were able to generate more mature biofilms on biomaterial surfaces. Additionally, c-di-GMP was found to negatively regulate bacterial swimming motility and enhance the ability to cope with environmental stresses. The results also reiterate the canonical function of c-di-GMP, which is to reduce the motility of bacteria. Concurrently, gene expression analysis confirmed these findings, revealing that genes related to motility (*flhC*, *flhD*, *motA*, *motB*, *ycgR*), extracellular polymeric substances (EPS) synthesis (*csgA*, *csgD*, *bcsA*, *ynfM*), and stress resistance (*sodA*, *katE*, *rstA*, *ibpA*, *ibpB*, *hdeA*, *hdeD*, *gadA*, *gadB*) were consistently up-regulated in O*dgcQ* with high c-di-GMP levels. Importantly, Δ*dgcQ* considerably promoted the adhesion to and invasion of host cells and elicited a stronger host immune response, whereas O*dgcQ* impaired the ability to interact with host cells, as evidenced by decreased adhesion/invasion and inhibited release of inflammatory cytokines (IL-1β, IFN-β, IP-10, and NF-κB). Collectively, our findings shed light on the c-di-GMP signaling pathway's role in BAIs and propose that modulating this pathway could be a promising strategy for combating *E. coli*-induced BAIs.

## 1 Introduction

Commensal *Escherichia coli* (*E. coli*) is not pathogenic under normal intestinal conditions. However, once it is transported across a compromised intestinal barrier (e.g., shock, trauma, or chronic disease [1–3]), it becomes a clinically important opportunistic pathogen, particularly in immunocompromised patients with implanted biomaterials. In the clinical environment, biomaterial-associated infections (BAIs) are one of the most common and severe complications [4,5]. In the USA, BAIs account for 25.6% of all hospital-associated infections [6]. These infections are particularly difficult to eradicate because biofilms on medical devices provide protection against host immune clearance and confer resistance to antibiotic therapy [7]. Notably, nosocomial infections caused by *E. coli* have shown a concerning increase from 10% in 2007 to 15% in 2014 [8,9]. This pathogen ranks among the most frequently isolated microorganisms in surgical implant-related infections, including catheter-associated urinary tract infections [10], central line-associated bloodstream infections [11], and ventilator-associated pneumonia [12].

The pathogenesis of BAIs centers on two critical processes: bacterial adhesion and biofilm formation [13]. Biomaterials act as substrates for initial bacterial adhesion, where *E. coli* transitions from reversible surface attachment (0–12 h) to irreversible colonization. Subsequent biofilm maturation (24–48 h) involves microcolony organization, extracellular polymeric substance (EPS) secretion, and 3D architectural assembly, forming protective microbial communities on implant surfaces [14]. Cyclic di-GMP (c-di-GMP) is a ubiquitous second messenger in Gram-negative bacteria, including *Pseudomonas aeruginosa*, *Pseudomonas putida*, *Vibrio vulnificus* and *E. coli*. This molecule is synthesized from two GTP molecules by diguanylate cyclases (DGCs, containing GGDEF domains) and degraded by phosphodiesterases (PDEs, featuring either EAL or HD-GYP domains) [15]. Substantial evidence indicates that c-di-GMP serves as the primary regulator of bacterial lifestyle switching from planktonic to sessile biofilm states [16,17]. In *E. coli* K-12, 29 genes encode proteins containing GGDEF and/or EAL domains, comprising 12 functional DGCs, 13 PDEs, and four enzymatically inactive “degenerate” proteins [18]. The *E. coli* ATCC25922 strain maintains an identical genetic repertoire for c-di-GMP metabolism. Among these components, *dgcQ* encodes a diguanylate cyclase (DgcQ) featuring both GGDEF and CHASE domains. Interestingly, inactivation of *dgcQ* in *E. coli* has been observed to enhance bacterial adhesion by increasing swimming motility [19]. In contrast, studies in other species (*Comamonas testosteroni* [20], *Pseudomonas fluorescens* [21], *Pseudomonas putida* [22], and *Pseudomonas syringa* [23]) demonstrate that heterologous expression of *dgcQ* elevates c-di-GMP levels and promotes mature biofilm formation. However, in these studies, the *dgcQ* gene was not expressed from its native promoter, and the experiments did not involve *E. coli*. Thus, we cannot precisely determine the phenotype and function it regulates in *E. coli*. These perplexing findings suggest that the modular enzymes involved in c-di-GMP regulation are more complex than anticipated. Moreover, we cannot simply equate BAIs with bacterial biofilms, as BAIs arise from intricate interactions among pathogens, biomaterials, and host immune responses [24].

Polyvinyl chloride (PVC) remains widely used in medical catheters due to its favorable material properties, availability, and low cost [25]. Unfortunately, PVC-associated infections pose significant clinical challenges. Comparative studies reveal that PVC endotracheal tubes exhibit higher susceptibility to bacterial adhesion than silicone surfaces, which correlates with increased ventilator-associated pneumonia risk in intubated patients [26]. However, the effects of c-di-GMP in PVC-associated infections caused by *E. coli* remains underexplored. Consequently, in-depth research on the role of c-di-GMP in PVC BAIs caused by *E. coli* not only aids in understanding the mechanism of bacterial biofilm formation but also offers theoretical and practical guidance for developing more effective antibacterial materials.

In the present study, we constructed four mutant strains of *E. coli* ATCC25922 with varying levels of c-di-GMP by knocking out or overexpressing *dgcQ* to elucidate the contribution of c-di-GMP to PVC BAIs. We systematically investigated the effects of c-di-GMP on biological functions including growth, motility, and environmental stress resistance in planktonic bacteria. Using PVC substrates, we dynamically monitored biofilm formation processes and analyzed their morphology and ultrastructure. Subsequently, we investigated the changes in gene expression in biofilm bacteria. We further examined the effects of c-di-GMP on *E. coli*'s adhesion and invasion capabilities, and on host-pathogen interactions. Collectively, our findings advance the understanding of molecular mechanisms underlying *E. coli*-mediated medical device infections and suggest that targeting c-di-GMP could be a promising strategy for preventing biofilm formation and reducing the risk of BAIs.

## 2 Materials and methods

### 2.1 Bacterial strains, plasmids, and growth conditions

The strains and plasmids used in the present study are listed in Table 1. *E. coli* ATCC25922 was obtained from the Institute of Microbiology, Chinese Academy of Sciences. Plasmids pCas, pTargetF, and pBAD30 were purchased from Haijihaoge Biotech Co., Ltd. (Shanghai, China). All strains were routinely cultured at 37°C in Luria-Bertani (LB) broth. When required, the following supplements were added: ampicillin (100 μg/mL), kanamycin (50 μg/mL), spectinomycin (50 μg/mL), and L-arabinose.

**Table 1:** Bacterial strains and plasmids used in this study.

Strains and plasmids	Description	Source
*E. coli* ATCC25922	Wild-type	CGMCC
*E. coli* ATCC25922Δ*dgcQ*	In frame deletion of *dgcQ* derived from ATCC25922	This study
WT	*E. coli* ATCC25922 containing an expression vector pBAD30, Amp^r^	This study
Δ*dgcQ*	*E. coli* ATCC25922Δ*dgcQ* containing an expression vector pBAD30, Amp^r^	This study
CΔ*dgcQ*	*E. coli* ATCC25922Δ*dgcQ* containing an expression vector pBAD-*dgcQ*, Amp^r^	This study
O*dgcQ*	*E. coli* ATCC25922 containing an expression vector pBAD-*dgcQ*, Amp^r^	This study
*E. coli* DH5a	*E. coli* for cloning	Tsingke
pCAS	*repA101*(Ts) *kan Pcas-cas9 ParaB-Red lacIq Ptrc-sgRNA-pMB1*	Haijihaoge
pTargetF	*pMB1 aadA* sgRNA	Haijihaoge
pTargetF-*dgcQ*	*pMB1 aadA* sgRNA*-dgcQ*, sgRNA*-dgcQ*	This study
pBAD30	Expression vector	Haijihaoge
pBAD-*dgcQ*	pBAD30 with 1695 bp *dgcQ* gene	This study

### 2.2 Construction of *E. coli* mutant strains

The *dgcQ* gene knockout mutant ATCC25922Δ*dgcQ* was constructed using the pCas/pTargetF CRISPR-Cas9 system. Specifically, donor DNA fragments were amplified from the *E. coli* ATCC25922 genome: the upstream fragment was amplified using primers P1/P2, and the downstream fragment was amplified using primers P3/P4. Following purification, these fragments were fused by overlapping PCR to generate donor DNA. Two sgRNAs targeting *dgcQ* were subsequently cloned into pTargetF to generate pTargetF-*dgcQ.* Both pTarget-*dgcQ* and donor DNA were electroporated into ATCC25922 cells harboring pCas plasmid. Positive clones were selected on LB plates supplemented with kanamycin (50 μg/mL) and spectinomycin (50 μg/mL). To eliminate plasmids, selected colonies were streaked onto LB agar with kanamycin (50 μg/mL) and incubated at 30°C overnight to remove the pTargetF plasmid. Subsequently, colonies were incubated at 42°C overnight to remove the temperature-sensitive pCas plasmid.

For the *dgcQ*-overexpression mutant (O*dgcQ*), the *dgcQ* coding sequence was amplified with primers P1/P4, while pBAD30 vector was linearized by inverse PCR using primers P5/P6. These fragments were assembled using the ClonExpress® II One Step Cloning Kit (Vazyme Biotech Co., Ltd., Nanjing, China) to generate pBAD-*dgcQ.*

The pBAD30 plasmid was transformed into competent cells of ATCC25922 and ATCC25922Δ*dgcQ* to produce strains ATCC25922/pBAD30 (WT) and ATCC25922Δ*dgcQ*/pBAD30 (Δ*dgcQ*). The pBAD-*dgcQ* plasmid was transformed into competent cells of ATCC25922Δ*dgcQ* and ATCC25922 to generate complemented strain ATCC25922Δ*dgcQ*/pBAD-*dgcQ* (CΔ*dgcQ*) and overexpression strain ATCC25922/pBAD-*dgcQ* (O*dgcQ*). All mutant strains were verified through PCR amplification using 2 × Phanta® Max Master Mix (Dye Plus; Vazyme Biotech Co., Ltd., Nanjing, China), 1% agarose gel electrophoresis, and Sanger sequencing. Primer sequences are provided in S1 Table.

### 2.3 Expression of *dgcQ* in the four mutant strains

To investigate *dgcQ* expression in WT, Δ*dgcQ*, CΔ*dgcQ*, and O*dgcQ* strains, overnight cultures of each strain were sub-cultured (1:100) in 10 mL LB broth supplemented with 100 μg/mL ampicillin, followed by incubation at 37°C with 220 rpm shaking. When the OD_600_ reached 0.4 ~ 0.6, 0.2% L-arabinose was added to induce gene expression, with parallel control cultures maintained without inducer. All cultures were further incubated for 16 h under identical conditions. Cells were harvested by centrifugation (4,000 rpm, 10 min, 4°C) and washed twice with ice-cold PBS. Total RNA was extracted using TRIzol^TM^ reagent (Life Technologies, USA) according to the previously described method [27]. RNA integrity was verified by 1% agarose gel electrophoresis. First-strand cDNA synthesis was performed using the HiScript II Q RT SuperMix for qPCR (Vazyme Biotech Co., Ltd., Nanjing, China). Quantitative real-time PCR (qRT-PCR) was conducted on an Applied Biosystems 7500 system with SYBR Green Master Mix (Vazyme Biotech Co., Ltd., Nanjing, China). The *dgcQ* expression levels were normalized to *16S rRNA* using the 2^-∆∆Ct^ method.

### 2.4 DgcQ expression in the four mutant strains

To determine the optimal induction concentration of pBAD-*dgcQ* plasmid, O*dgcQ* cultures were subcultured (1:100) as previously described. When the OD_600_ reached 0.4 ~ 0.6, gradient concentrations of L-arabinose (0.05%, 0.1%, 0.2%, 0.4%) were added to each O*dgcQ* culture, which was then incubated for 16 h at 37°C with 220 rpm shaking. For comparative analysis of DgcQ expression in WT, Δ*dgcQ*, CΔ*dgcQ*, and O*dgcQ* strains, cultures were prepared as described above. When the OD_600_ reached 0.4 ~ 0.6, 0.2% L-arabinose was added and the cultures were incubated for 16 h at 37°C with 220 rpm shaking. After incubation, all cultures were harvested, washed twice with ice-cold PBS, and resuspended in 1 mL lysis solution (lysis buffer: 988 μL; protease inhibitor mixture: 1 μL; 1M DDT: 1 μL; 100 mM PMSF: 10 μL) at 4°C for 10 min. Ultrasonication (300 W, 10 s on/10 s off cycles, 20 min total) was performed to disrupt cells until lysate clarification. The lysate was centrifuged (12,000 rpm, 10 min, 4°C) to collect total protein. Total protein concentration was determined using a BCA protein assay kit. 30 μg protein was loaded and resolved on 10% SDS-PAGE, followed by electrophoretic transfer to PVDF membranes. After blocking in 5% skim milk for 1 h, membranes were incubated with anti-DgcQ antibody (1:2,000) at 4°C for 16 h, washed three times with TBST. Subsequently, membranes were incubated with HRP-conjugated secondary antibody (1:4,000) at room temperature for 1 h, washed three times with TBST. Immunoreactive bands were visualized using an ECL system. Anti-GAPDH (1:5,000) served as loading control. All antibodies were provided by Cusabio Biotech Co., Ltd. (Wuhan, China). Parallel analyses were performed for ATCC25922 and ATCC25922Δ*dgcQ* using identical protocols.

### 2.5 Quantitation of cellular c-di-GMP in the four strains

To assess the regulatory role of *dgcQ* on c-di-GMP homeostasis, intracellular c-di-GMP levels were quantified in the WT, Δ*dgcQ*, CΔ*dgcQ*, and O*dgcQ* strains. Cultures were grown under standard conditions (37°C, 220 rpm) in LB broth supplemented with 100 μg/mL ampicillin. When the OD_600_ reached 0.4 ~ 0.6, 0.2% L-arabinose was supplemented to induce gene expression, followed by 16 h incubation. After incubation, the cultures were harvested by centrifugation, washed twice with ice-cold PBS, and subjected to c-di-GMP extraction as previously described [28]. Quantification was performed using a commercial c-di-GMP ELISA kit (Meimian Biotech Co., Ltd., Jiangsu, China) according to the manufacturer's protocol. Cellular c-di-GMP levels were normalized to total bacterial protein and expressed as pmol/g bacterial protein. Three independent biological replicates were analyzed. Parallel measurements were conducted for ATCC25922 and ATCC25922Δ*dgcQ* using identical protocols.

### 2.6 Planktonic growth curve

Bacterial growth curves were generated as previously described [29]. Briefly, the WT, Δ*dgcQ*, CΔ*dgcQ*, and O*dgcQ* strains were cultured in 100 mL LB broth supplemented with 100 μg/mL ampicillin with or without 0.2% L-arabinose and incubated at 37°C for 48 h with shaking at 220 rpm. The OD_600_ values of the four strains were measured periodically using a spectrophotometer. The experiments were conducted three times independently.

### 2.7 Biofilm biomass on PVC biomaterials

The crystal violet (CV) staining assay was performed to evaluate biofilm biomass on PVC substrates following a previously described method with minor modifications[30]. Briefly, overnight cultures of WT, Δ*dgcQ*, CΔ*dgcQ*, and O*dgcQ* were normalized to an initial OD_600_ of 0.4 using fresh LB broth containing 100 μg/mL ampicillin supplemented with 0.2% L-arabinose. A sterile 5 × 5 mm PVC piece (Kewei Co., Ltd., Guangzhou, China) and 100 μL bacterial suspension were added to each well of a 96-well plate, followed by static incubation at 37°C for 12, 24, 48, and 72 h. Four to six technical replicates per strain were included in each plate. After incubation, the PVC pieces were gently washed twice with PBS to remove non-adherent bacteria, stained with 0.1% crystal violet solution for 20 min at room temperature, and subsequently washed three times with PBS. Bound dye was solubilized in 95% ethanol, and absorbance at 570 nm was quantified using a microplate reader. The experiments were conducted three times independently. All experiments included PVC substrates pre-treated with human plasma (healthy donor-derived, 37°C, 4 h) after sterilization.

### 2.8 Viability of bacteria in biofilms on PVC biomaterials

The tetrazolium salt (XTT) reduction assay was performed to evaluate the bacteria viability in biofilms using the XTT Bacteria Proliferation and Cytotoxicity Kit (KeyGEN Biotech Co., Ltd., Nanjing, China). Biofilms were established in 96-well plates containing sterile 5 × 5 mm PVC pieces following the protocol described in Section 2.7. Four to six wells per strain were inoculated in each plate. After incubation for 12–72 h at 37°C, PVC pieces were gently washed with PBS to remove non-adherent bacteria. Each well was supplemented with 100 μL of fresh LB broth and 20 μL XTT solution, followed by incubation at 37°C in the dark for 2 h. Absorbance at 450 nm was measured using a microplate reader. The experiments were conducted three times independently.

### 2.9 Swimming and swarming motility

WT, Δ*dgcQ*, CΔ*dgcQ*, and O*dgcQ* strains were cultured in LB broth at 37°C with 220 rpm shaking for 16 h. Cultures were harvested by centrifugation (4,000 rpm, 10 min, 4°C) and resuspended in sterile PBS to normalize the OD_600_ to 1.0. Swimming motility plates (0.3% agar containing 1% tryptone, 0.25% NaCl, 0.01% ampicillin, 0.2% L-arabinose) and swarming motility plates (0.4% agar with identical components) were prepared. Bacterial suspensions (2 μL) were spot-inoculated at the center of each plate using a sterile pipette tip. Plates were incubated at 37°C for 6–24 h, and motility diameters were measured with a digital caliper. The experiments were conducted three times independently.

### 2.10 Morphology of biofilms on PVC biomaterials

The morphology of WT, Δ*dgcQ*, and O*dgcQ* biofilms on PVC was analyzed using confocal laser scanning microscopy (CLSM). Briefly, overnight cultures of WT, Δ*dgcQ*, and O*dgcQ* were adjusted to an initial OD_600_ of 0.4 using fresh LB broth containing 100 μg/mL ampicillin supplemented with 0.2% L-arabinose. Bacterial suspensions (2 mL) and 10 × 10 mm PVC pieces were co-cultured in 24-well plates at 37°C for 12–72 h. After incubation, PVC pieces were gently washed with PBS to remove non-adherent bacteria, stained with a SYTO9/propidium iodide (PI) mixture (Live/Dead® BacLight^TM^ Bacterial Viability Kit, Life Technologies, USA) for 20 min at 25°C in the dark, and rinsed with normal saline to remove unbound dye. CLSM imaging was performed on a Leica TCS SP2 system (Leica Microsystems, Germany). Five random fields per sample were acquired through Z-axis stack scanning. Biofilm thickness was measured by optical sectioning from the substratum to the biofilm surface, while live cell proportions were calculated based on SYTO9 (green)/PI (red) fluorescence intensity ratios using ImageJ v1.53 with the Biovolume plugin.

### 2.11 Ultrastructure of biofilms on PVC biomaterials

The ultrastructure of WT, Δ*dgcQ*, and O*dgcQ* biofilms on PVC was analyzed by scanning electron microscopy (SEM) as described in our previous study [30]. Briefly, biofilm samples were prepared following the method described in CLSM protocol. After removing non-adherent bacteria, PVC pieces were fixed in 2% glutaraldehyde at 4°C for 16 h. The samples were sequentially subjected to primary fixation, post-fixation 1% osmium tetroxide, dehydration with gradient ethanol, and permeation with tert-butanol. Following critical point drying with CO_2_ and ion sputter coating with golden brown, the samples were imaged using an S-3000N scanning electron microscope (Hitachi, Japan) at 20 kV and 20,000 × magnification. Three biological replicates were imaged per strain.

### 2.12 Bacterial adaptation to environmental stress

Bacterial adaptation to environmental stress was examined as previously described [31,32]. For oxidative/acid stress assays, overnight cultures of WT, Δ*dgcQ*, and O*dgcQ* were harvested and adjusted to an OD_600_ of 1.0. Bacterial suspensions were exposed to LB broth containing 5 mM H_2_O_2_ or LB broth acidified to pH 4.0/3.0 with HCl at 37°C for 30 min. For heat stress, bacterial suspensions were incubated in a 48°C water bath for 20 min. After treatments, viable cells were quantified by plating on LB agar plates at serial dilutions. After gradient dilution, viable cells were counted by colony-forming units (CFU) on LB agar plates after 24 h incubation at 37°C. Survival rates were calculated as: (CFU post-treatment/ CFU pre-treatment) × 100%. The experiments were conducted three times independently.

### 2.13 Gene expression in biofilms on PVC biomaterials

Overnight cultures of WT, Δ*dgcQ*, and O*dgcQ* were prepared as described above. Bacterial suspensions (20 mL) and sterile 6 × 6 cm PVC pieces were co-cultured in 90 mm cell culture dishes at 37°C for 48 h. After co-culture, PVC pieces were gently washed with PBS to remove non-adherent bacteria. Biofilms on PVC pieces were scraped using sterile cell scrapers and collected in 2 mL microcentrifuge tubes. Total RNA was extracted and reverse-transcribed into cDNA. Flagellar motility genes (*flhC*, *flhD*, *motA*, *motB*, *ycgR*), extracellular polymeric substance (EPS) synthesis genes (*csgA*, *csgD*, *bcsA*, *ynfM*), and stress response genes (*sodA*, *katE*, *rstA*, *ibpA*, *ibpB*, *hdeA*, *hdeD*, *gadA*, *gadB*) were quantified using qRT-PCR. *16S rRNA* served as the endogenous control. WT biofilm samples were designated as calibrators. The 2^−∆∆Ct^ method was used to analyze qRT-PCR data. Primer sequences are listed in S2 Table. The experiments were conducted three times independently.

### 2.14 Host-pathogen interaction

#### 2.14.1 Co-culture and infection.

WT, Δ*dgcQ*, CΔ*dgcQ*, and O*dgcQ* strains were cultured in LB broth at 37°C with 220 rpm shaking for 18 h. Bacterial suspensions were adjusted to an OD_600_ of 1.0 and stained with SYTO9^®^Green Fluorescent Dye for 15 min in the dark. The HCT116 human intestinal epithelial cell line was kindly donated by Prof. Li (Department of Colorectal Surgery, Yunnan Cancer Hospital, Yunnan, China). HCT116 cells were cultivated in RPMI-1640 medium supplemented with 10% fetal bovine serum (FBS; Gibco) under 5% CO_2_ at 37°C.

For bacterial infection, sterile 20 × 20 mm PVC pieces were placed in 6-well plates, and HCT116 cells were seeded at a density of 1 × 10^6^ cells/well. When cells reached 80% confluency, each well was washed twice with ice-cold PBS, and fresh RPMI-1640 medium containing 100 μg/mL ampicillin and 2 mg/mL L-arabinose was added. Subsequently, HCT116 cells were challenged with WT, Δ*dgcQ*, CΔ*dgcQ*, or O*dgcQ* at a multiplicity of infection (MOI) of 100 (bacteria:cells = 100:1) at 37°C under 5% CO_2_. After 4 h of infection, the 6-well plates were observed directly using a fluorescence microscope. After 16 h of infection, the plates were observed following PBS washing to remove non-adherent bacteria.

#### 2.14.2 Adhesion and invasion to host cells.

For adhesion assays, HCT116 cells (1 × 10^6^ cells/well) along with sterile PVC pieces were seeded and cultured in 6-well plates as previously described. Cells were infected with WT, Δ*dgcQ*, CΔ*dgcQ*, or O*dgcQ* in RPMI-1640 containing 100 μg/mL ampicillin and 2 mg/mL L-arabinose at a MOI of 100. After incubation for 2 h, non-adherent bacteria were removed by gently washing three times with ice-cold PBS. HCT116 cells were lysed with 0.2% Triton X-100 for 10 min. After gradient dilution, lysates were plated onto LB agar plates, and CFUs were counted after 18 h incubation at 37°C. The experiments were conducted seven times independently.

For invasion assays, following the 2 h infection protocol, extracellular bacteria were eliminated by treatment with 100 μg/mL gentamicin (1 h, 37°C). Cells were washed with ice-cold PBS three times to remove gentamicin, lysed, and processed as above. Intracellular bacterial load was quantified via CFU enumeration. The experiments were conducted seven times independently.

#### 2.14.3 Quantification of cytokines in the co-culture system.

HCT116 cells (1 × 10^6^ cells/well) along with sterile PVC pieces were seeded and cultured in 6-well plates as previously described. Following co-culture with WT, Δ*dgcQ*, and O*dgcQ* at an MOI of 100 for 3 h, cytokines IL-1β and IFN-β in the culture supernatants, as well as NF-κB and IP-10 in HCT116 cell lysates, were quantified by ELISA. Uninfected HCT116 cells served as negative controls. HCT116 cells infected with WT strain served as the positive controls. Human IL-1β, IFN-β, NF-κB, and IP-10 ELISA kits (Cusabio Biotech Co., Ltd., Wuhan, China) were used according to the manufacturer's protocol. The experiments were conducted three times independently.

### 2.15 Statistical analysis

Statistical analyses were performed using the IBM SPSS Statistics 23.0. All graphs were generated using GraphPad Prism 8.0.1 and Adobe Illustrator CC 2019. The experimental data that followed the normal distribution or that did so after conversion are expressed as the mean ± standard deviation. One-way analysis of variance (ANOVA) with LSD or Tamhane’s T2 or Tukeyʼs HSD multiple comparison tests were used for intergroup comparisons. The threshold for statistical significance was set at **P* *< 0.05.

## 3 Results

### 3.1 Verification of WT, Δ*dgcQ*, CΔ*dgcQ*, and O*dgcQ* strains

**Fig 1:**
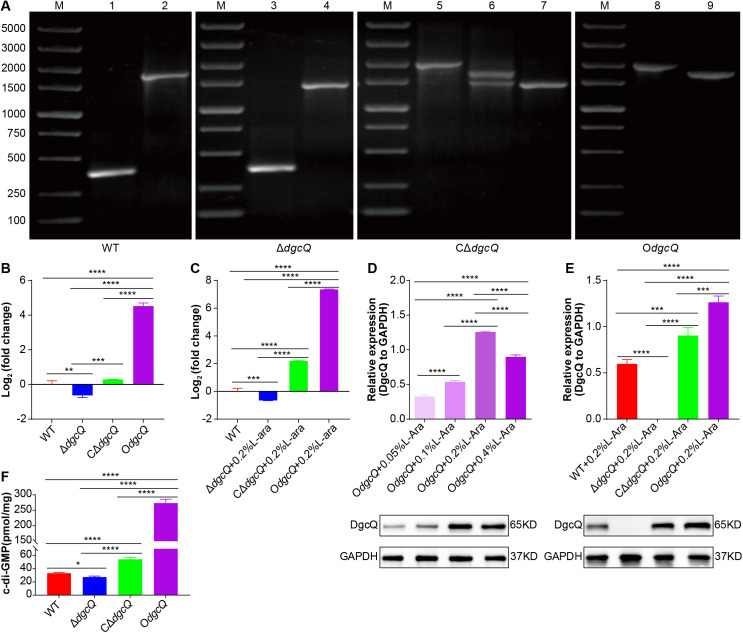
Construction and identification of WT, Δ*dgcQ*, C*dgcQ*, and O*dgcQ* strains. (A) PCR identification of the WT, Δ*dgcQ*, CΔ*dgcQ*, and O*dgcQ* strains. Lane1, 3, 5, 8 amplified by primers P7/8 (expected sizes: 378 bp, 378 bp, 1974 bp, 1974 bp). Lane2, 4, 6, 9 amplified by primers P1/4 (expected sizes: 1695 bp, 1425 bp, 1695/1425 bp, 1695 bp). Lane7 amplified by primers P1/9 (expected sizes: 1445 bp). M: 5000 bp ladder. (B) qRT-PCR identification of the *dgcQ* mRNA of WT, Δ*dgcQ*, CΔ*dgcQ*, and O*dgcQ* strains (n = 3). (C) qRT-PCR identification of the *dgcQ* mRNA of WT, Δ*dgcQ*, CΔ*dgcQ*, and O*dgcQ* strains induced by 0.2% L-arabinose (n = 3). (D) The DgcQ expression of O*dgcQ* strain induced by various concentrations of L-arabinose (n = 3). (E) The DgcQ expression of WT, Δ*dgcQ*, CΔ*dgcQ*, and O*dgcQ* strains induced by 0.2% L-arabinose (n = 3). (F) The c-di-GMP levels of WT, Δ*dgcQ*, CΔ*dgcQ*, and O*dgcQ* strains (n = 3). Statistical analysis was performed by One-way ANOVA with LSD (B, C, D, and E) or Tamhane’s T2 (F) multiple comparison test. The values obtained in the WT strain were used as control in qRT-PCR assays (B, C). The DgcQ protein levels were shown relative to GAPDH (D, E). ^*^P < 0.05; ^**^P < 0.01; ^***^P < 0.001; ^****^P < 0.0001. Error bars indicated standard deviations.

Four mutant strains were identified by PCR and agarose gel electrophoresis. The results demonstrated that WT, Δ*dgcQ*, CΔ*dgcQ*, and O*dgcQ* strains were successfully generated using the pCas/pTargetF and pBAD/*araC* expression systems (Fig 1A). *dgcQ* expression in these strains was confirmed by qRT-PCR (Fig 1B and 1C). Specifically, *dgcQ* expression was restored in CΔ*dgcQ* and upregulated in O*dgcQ* (Fig 1B). Following 0.2% L-arabinose induction, *dgcQ* expression levels in CΔ*dgcQ* and O*dgcQ* were markedly enhanced, with CΔ*dgcQ* exhibiting 4-fold higher expression compared to WT, and O*dgcQ* showing a 180-fold increase (Fig 1C). In contrast, *dgcQ* expression remained low in Δ*dgcQ* (Fig 1B and 1C).

Western blot analysis revealed that DgcQ expression in O*dgcQ* increased dose-dependently with L-arabinose concentrations up to 0.2%, but declined at 0.4% (Fig 1D). These data indicate that 0.2% L-arabinose is the optimal induction concentration. After induction, DgcQ was undetectable in Δ*dgcQ*, while CΔ*dgcQ* displayed intermediate expression levels between WT and O*dgcQ* (Fig 1E).

As shown in Fig 1F, the intracellular c-di-GMP concentration in O*dgcQ* was 273.3 pmol/g, higher than that in CΔ*dgcQ* (54.2 pmol/g). In contrast, c-di-GMP levels in Δ*dgcQ* were 27.3 pmol/g, representing a 17% decrease compared to WT (33.1 pmol/g). These findings conclusively establish DgcQ as a functional diguanylate cyclase regulating c-di-GMP homeostasis.

Identification of the ATCC25922 and ATCC25922Δ*dgcQ* strains is shown in S1 Fig. Results demonstrated that neither DgcQ protein levels nor c-di-GMP concentrations in *E. coli* were significantly affected by the presence or absence of the pBAD vector. This confirms that the phenotypic differences observed in our original experiments (conducted with the vector) resulted directly from *dgcQ* genetic manipulation and were not due to vector effects.

### 3.2 c-di-GMP affects biofilm formation on PVC biomaterials

**Fig 2:**
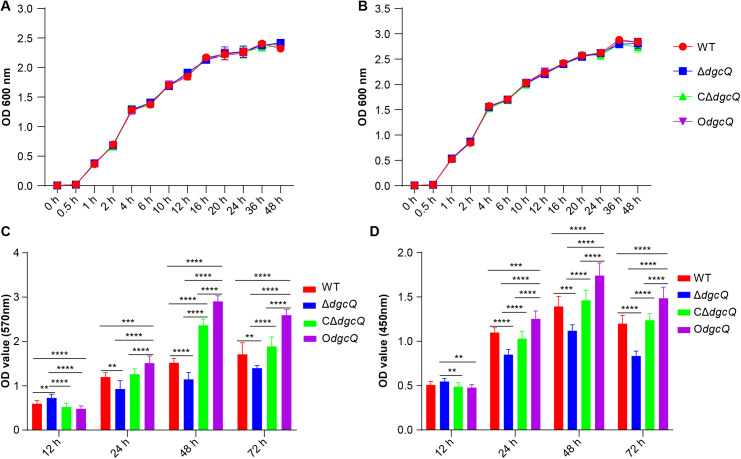
Biological characteristics analysis of the four mutant strains. (A) Growth curve of WT, Δ*dgcQ*, CΔ*dgcQ*, and O*dgcQ* strains as determined by optical density (OD 600 nm) (n = 3). (B) Growth curve of WT, Δ*dgcQ*, CΔ*dgcQ*, and O*dgcQ* strains induced by 0.2% L-arabinose as determined by optical density (OD 600 nm) (n = 3). (C) The biofilm biomass of WT, Δ*dgcQ*, CΔ*dgcQ*, and O*dgcQ* strains on PVC (n = 3). (D) The bacterial viability of WT, Δ*dgcQ*, CΔ*dgcQ*, and O*dgcQ* biofilms on PVC (n = 3). Statistical analysis was performed by One-way ANOVA with Tukey’s HSD multiple comparison test for all the assays. ^**^ **P* *< 0.01; ^***^ **P* *< 0.001; ^****^ **P* *< 0.0001. Error bars indicated standard deviations.

Growth curve analysis revealed comparable growth patterns among the four mutant *E. coli* strains in standard LB broth (Fig 2A). The addition of 0.2% L-arabinose to LB broth to induce differential DgcQ expression similarly showed no significant growth variations among the strains (Fig 2B).

Biofilm biomass analysis demonstrated temporal differences in PVC colonization, while *dgcQ* deletion (Δ*dgcQ*) transiently enhanced bacterial adhesion to PVC at 12 h (initial stage of biofilm formation). subsequent biofilm development from 24–72 h exhibited distinct patterns: O*dgcQ* biofilms accumulated the highest biomass, CΔ*dgcQ* showed restoration of WT characteristics, and Δ*dgcQ* consistently displayed the lowest colonization capacity (Fig 2C).

XTT assays revealed that dynamic viability profiles within biofilms. At 12 h, Δ*dgcQ* exhibited a modest but statistically significant viability increase compared to CΔ*dgcQ* (**P* *= 0.0081) and O*dgcQ* (*P* = 0.0013). From 24–72 h, the XTT results demonstrated a hierarchical viability: O*dgcQ* > CΔ**dgcQ* *= WT > Δ*dgcQ* (Fig 2D).

### 3.3 DgcQ represses the swimming ability of *E. coli* but not swarming

**Fig 3:**
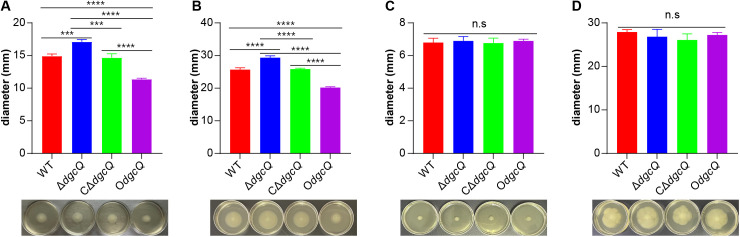
Comparison of the swimming and swarming ability among the four mutant strains. (A) Swimming ability at 6 h (n = 3). (B) Swimming ability at 12 h (n = 3). (C) Swarming ability at 12 h (n = 3). (D) Swarming ability at 24 h (n = 3). Statistical analysis was performed by One-way ANOVA with LSD multiple comparison test for all the assays. ^***^P < 0.001; ^****^P < 0.0001. n.s, no significance. Error bars indicated standard deviations.

Motility assays revealed distinct swimming phenotypes among the strains (Fig 3A and 3B). The Δ*dgcQ* exhibited significantly expanded motility zones compared to other strains at both 6 h (*P* < 0.001) and 12 h (*P* < 0.0001), with diameter measurements following this descending order: Δ**dgcQ* *> WT/CΔ**dgcQ* *>* *O*dgcQ*. These findings demonstrate that *dgcQ* deletion enhances flagellar-driven motility, whereas *dgcQ* overexpression substantially impairs bacterial swimming capacity. In contrast, swarming assays showed no statistically significant differences between strains at either 12 h or 24 h under identical experimental conditions (Fig 3C and 3D).

### 3.4 Morphology of biofilms on PVC biomaterials

**Fig 4:**
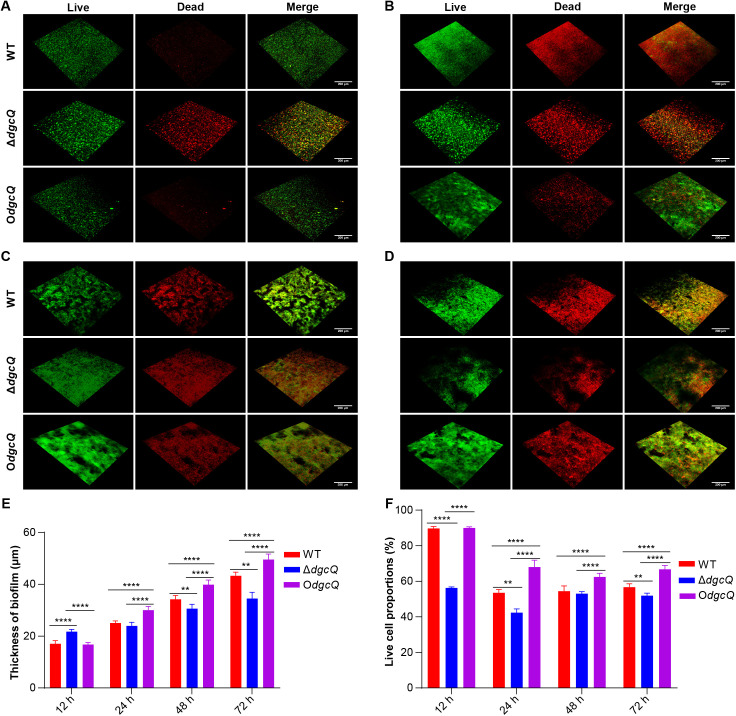
Thickness and live cell proportions of biofilms on PVC observed by CLSM. (A) Observation at 12 h. (B) Observation at 24 h. (C) Observation at 48 h. (D) Observation at 72 h. (E) The thickness of biofilms at 12, 24, 48, 72 h (n = 5). (F) The live cell proportions of biofilms at 12, 24, 48, 72 h (n = 5). Green represented live cell, red represented dead cell. Scale bar: 200 μm. Statistical analysis was performed by One-way ANOVA with LSD multiple comparison test for all the assays. ^**^P < 0.01; ^****^P < 0.0001. Error bars indicated standard deviations.

Biofilm morphology was characterized through fluorescence-based viability staining following PVC co-culture periods (12, 24, 48, and 72 h). Live and dead bacteria in biofilms on PVC were differentially visualized using green (SYTO9) and red (PI) fluorescence markers, respectively (Fig 4A–4D). Quantitative morphometric analysis revealed temporal dynamics in biofilm development: Δ*dgcQ* exhibited the thickest biofilm at 12 h, but its biofilm formation rate slowed significantly from 24–72 h. In contrast, O*dgcQ* demonstrated accelerated biofilm maturation, maintaining superior thickness compared to WT and Δ*dgcQ* throughout 24–72 h (Fig 4E). Meanwhile, live/dead cell proportion analysis showed that the proportion of live bacteria in Δ*dgcQ* was consistently low during the experimental period, whereas WT and O*dgcQ* maintained higher proportions of live bacteria (Fig 4F).

### 3.5 Ultrastructure of biofilms on PVC biomaterials

**Fig 5:**
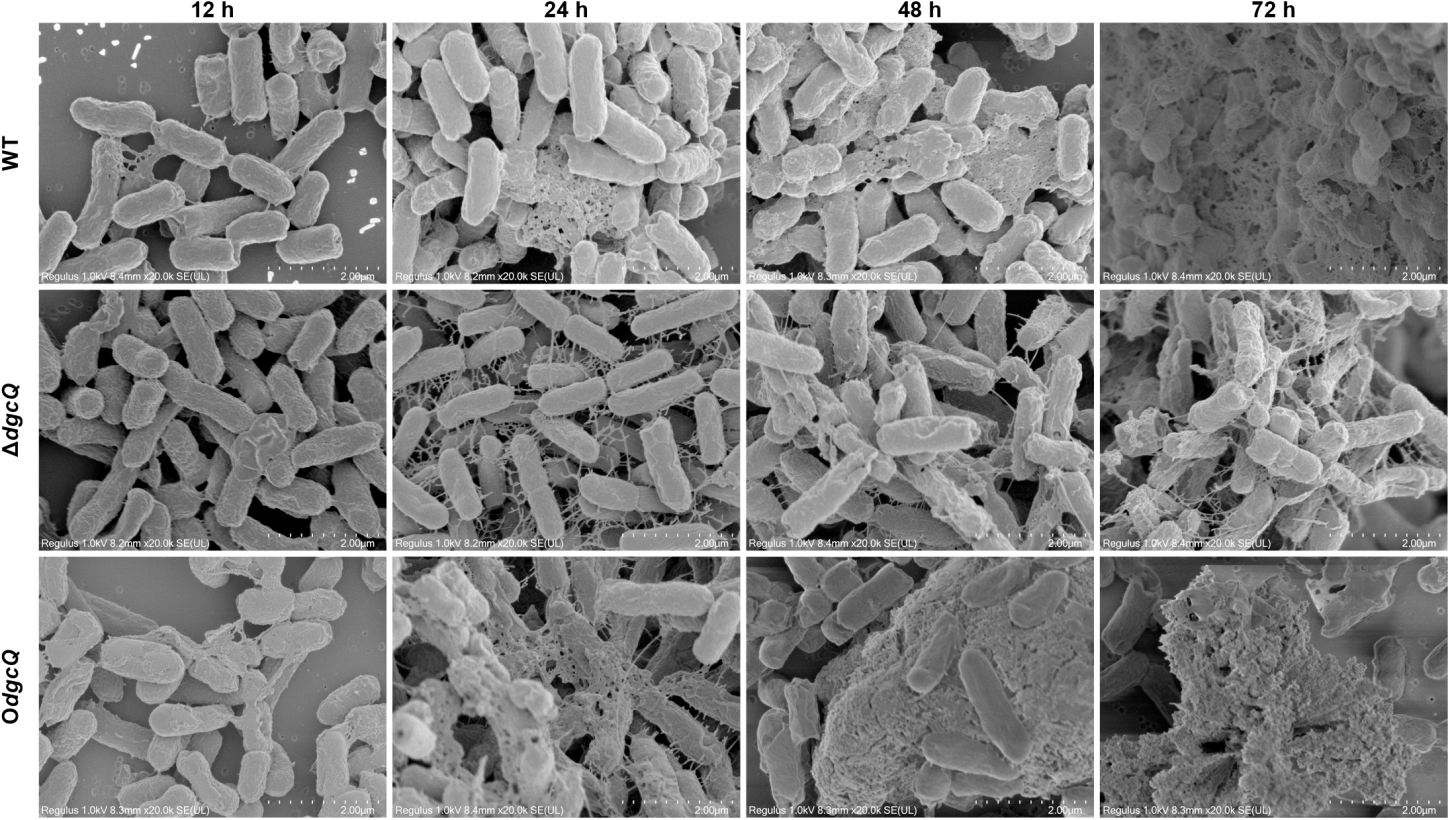
Microstructure of biofilms on PVC observed by SEM at 12, 24, 48, and 72 h. Scale bar: 2.0 μm.

Biofilm ultrastructure was analyzed by SEM following PVC co-culture. As demonstrated in Fig 5, biofilm architecture progressively increased in complexity, density, and three-dimensional organization with extended co-cultivation time across all *E. coli* strains. At the initial attachment phase (12 h), biofilms exhibited simple two-dimensional arrangements with minimal EPS deposition between cells. Bacterial aggregates were visible on PVC surfaces in all strains, though Δ*dgcQ* formed notably denser stacked aggregates compared to WT and O*dgcQ*. During maturation phases (24–72 h), Δ*dgcQ* biofilms remained structurally compromised, exhibiting sparse cellular networks with only grid-like intercellular connections and persistently low EPS accumulation. WT biofilms developed increasingly intricate three-dimensional matrices with moderate EPS deposition. O*dgcQ* biofilms displayed exceptional structural consolidation, forming densely packed architectures where bacterial cells were fully embedded within abundant EPS. These complexes developed into highly organized clusters with sophisticated three-dimensional topography.

### 3.6 DgcQ-mediated c-di-GMP signalling promotes *E. coli* adaptation to environmental stresses and alters gene expression in biofilms

**Fig 6:**
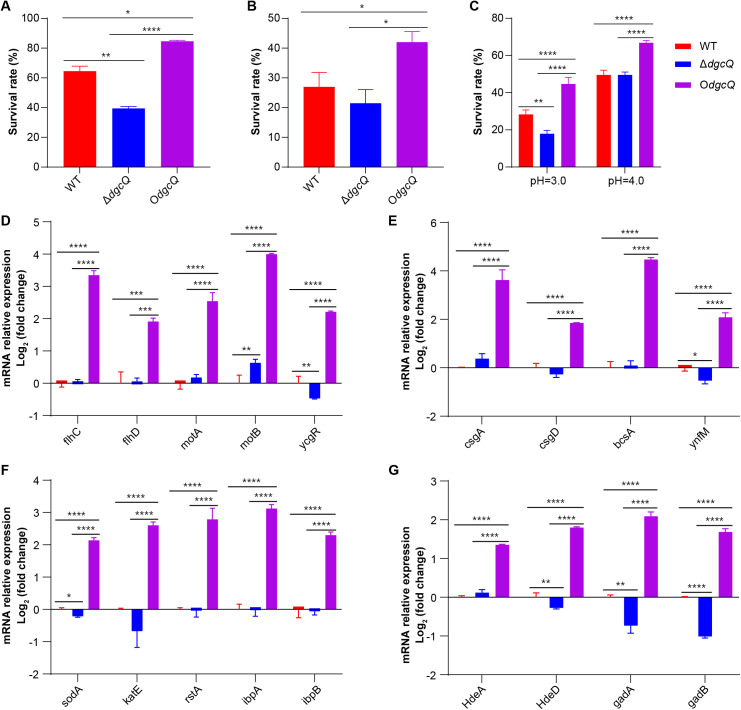
The adaptation to environmental stresses and related gene expression. (A) The adaptation to oxidative stress (n = 3). (B) The adaptation to heat stress (n = 3). (C) The adaptation to acid stress (n = 3). (D) Expression of genes related to motility (n = 3). (E) Expression of genes related to EPS (n = 3). (F) Expression of genes related to oxidative and heat stress (n = 3). (G) Expression of genes related to acid stress (n = 3). Statistical analysis was performed by One-way ANOVA with LSD multiple comparison test for all the assays. ^*^P < 0.05; ^**^P < 0.01; ^***^P < 0.001; ^****^P < 0.0001. The values obtained in the WT strain were used as control in qRT-PCR assays (D-G). Error bars indicated standard deviations.

The effects of c-di-GMP on the adaptation of *E. coli* to environmental stresses, including oxidative stress (Fig 6A), heat stress (Fig 6B), and acid stress (Fig 6C), were systematically evaluated. No significant differences were observed in the initial bacterial counts of WT, Δ*dgcQ*, and O*dgcQ* strains prior to stress exposure. Survival assays demonstrated significantly enhanced stress tolerance in O*dgcQ*, whereas Δ*dgcQ* exhibited compromised survival rates compared to WT under all tested conditions.

qRT-PCR was performed to identify the expression of flagellar motility genes (*flhC*, *flhD*, *motA*, *motB*, *ycgR*), EPS synthesis genes (*csgA*, *csgD*, *bcsA*, *ynfM*), oxidative stress response genes (*sodA*, *katE*, *rstA*), heat stress response genes (*ibpA*, *ibpB*), and acid stress response genes (*hdeA*, *hdeD*, *gadA*, *gadB*) in the WT, Δ*dgcQ*, and O*dgcQ* biofilms. Results showed that all of these genes were significantly upregulated in O*dgcQ*. In contrast, some genes in Δ*dgcQ* biofilms (including *ycgR*, *ynfM*, *sodA*, *hdeD*, *gadA*, and *gadB*) were significantly downregulated, while *motB* expression was increased (Fig 6D–6G).

### 3.7 c-di-GMP modulates *E. coli*-host interactions and suppresses inflammatory responses

**Fig 7:**
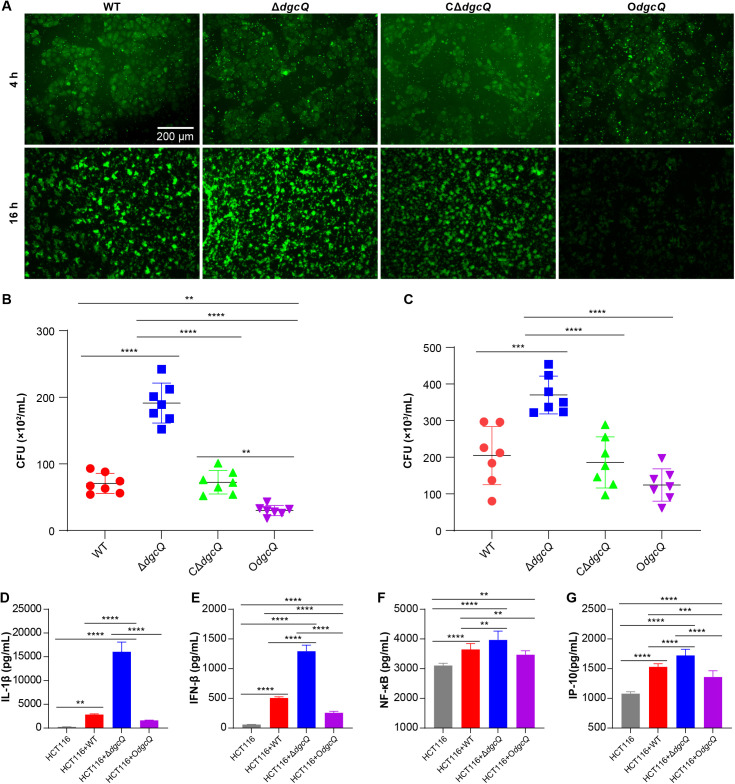
The adherence and invasion of the mutant strains to HCT116 and inflammatory response. (A) Observation of the co-culture system of the four mutant strains and HCT116 cells by fluorescence microscope. Green represented *E. coli*. Scale bar: 200 μm. (B) The adherence of *E. coli* to HCT116 (n = 7). (C) The invasion of *E. coli* to HCT116 (n = 7). (D) ELISA of IL-1β (n = 3). (E) ELISA of IFN-β (n = 3). (F) ELISA of NF-κb (n = 3). (G) ELISA of IP-10 (n = 3). Statistical analysis was performed by One-way ANOVA with LSD (D, E, and G) or Tamhane’s T2 (F) multiple comparison test. ^**^P < 0.01; ^***^P < 0.001; ^****^P < 0.0001. Error bars indicated standard deviations.

The modulatory effects of c-di-GMP on *E. coli* pathogenesis were evaluated through host-pathogen interaction assays. As demonstrated in Fig 7A–7C, Δ*dgcQ* exhibited significantly enhanced adhesion and invasion capacity toward HCT116 cells compared to WT, whereas O*dgcQ* showed attenuated infectivity, confirming an inverse relationship between c-di-GMP levels and bacterial virulence.

Host inflammatory responses were quantified via ELISA. The results (Fig 7D–7G) revealed that HCT116 cells infected with Δ*dgcQ* displayed the highest upregulation of IL-1β, IFN-β, NF-κB, and IP-10 levels. In contrast, cells infected with O*dgcQ* exhibited attenuated cytokine profiles, with all measured markers showing significantly lower levels. These findings suggest that c-di-GMP may mediate immunosuppression.

## 4 Discussion

Nosocomial infections persist as a major clinical challenge, with bacterial biofilms implicated in 60–70% of cases [33]. Our study establishes *dgcQ*-mediated c-di-GMP signaling as a mater regulator of *E. coli* biofilm dynamics on PVC biomaterials. We demonstrate that c-di-GMP functions as a precise “biofilm chronometer”, orchestrating a critical balance between early colonization and mature biofilm resilience. Crucially, this pathway dictates host-pathogen interactions by modulating both bacterial virulence and immune evasion. These data offers novel insights for targeted anti-infective strategies.

While Δ*dgcQ* (low c-di-GMP) exhibited enhanced initial adhesion to PVC surfaces (12 h), this aligns with its elevated swimming motility (Fig 3A and 3B). The results of initial adhesion and swimming ability seemed contradictory to each other. In fact, the paradoxical phenotypes can be explained by the dual role of low c-di-GMP in promoting motility-driven surface exploration while simultaneously increasing extracellular DNA (eDNA) production for attachment stabilization. These synergistic effects likely drove the transient biofilm advantage of Δ*dgcQ* during initial colonization (12 h), evidenced by thicker biofilm biomass (Fig 2C) and higher viability (Fig 2D). However, beyond this initial stage, O*dgcQ* (high c-di-GMP) formed structurally complex biofilms with dense EPS matrices at 24–72 h (Figs 4 and 5), supported by upregulation of EPS synthesis genes such as *csgD*, *csgA*, *bcsA*, and *ynfM* (Fig 6E). *csgD* acts as a transcription factor that binds to the promoter of the *csgBAC* operon, which encodes the major curli subunit *csgA* and provides positive feedback for c-di-GMP, promoting *bcsA* expression in some strains. *csgA* and *bcsA* are major biofilm regulators that respectively control the expression of curli and cellulose [34]. Concurrently, O*dgcQ* biofilms demonstrated significant upregulation of flagellar genes (*flhC/D*, *motA/B*) despite the established role of c-di-GMP in motility suppression through YcgR-mediated flagellar braking [35,36]. This implies a compensatory regulatory mechanism by which bacterial cells counteract motility inhibition through flagellar gene overexpression, revealing the complexity of c-di-GMP signaling networks.

XTT/CLSM analysis revealed that the viability of Δ*dgcQ* biofilms was superior to that of WT and O*dgcQ* biofilms at 12 h (Fig 2D), but the proportion of live bacteria was still lower (Fig 4A). This seemingly contradictory result of higher viability alongside a lower proportion of live bacteria in Δ*dgcQ* biofilms can be explained by the higher biofilm biomass at 12 h, as shown in Fig 2C. In contrast, the higher viability and live bacteria proportions of O*dgcQ* biofilms on PVC at the mature stages (24–72 h) indicated the long-term survival ability was enhanced in O*dgcQ* biofilms, correlating with significantly upregulated stress-response genes in O*dgcQ* biofilms (Fig 6F and 6G). To better elucidate the relation between *E. coli* adaptation to stresses and c-di-GMP levels, we further observed that O*dgcQ* with elevated c-di-GMP exhibited stronger tolerances to oxidative, heat, and acid stresses in the planktonic state*.* These findings further indicate that *E. coli* with elevated c-di-GMP are more prone to long-term survival under extreme environmental conditions. Previous studies showed that *csgD* plays an important role in enhancing the long-term survival and persistence of *Salmonella* in biofilms under stressful conditions [37]. Additionally, it has been found that c-di-GMP can downregulate bacterial metabolism by competing with RpoS/RpoD for promoter binding sites and can also directly reprogram bacterial metabolism in an RpoS-independent manner [38,39]. The low metabolic state of bacteria in mature biofilms is a consequence of a c-di-GMP-regulated survival strategy [40]. Therefore, we propose that elevated c-di-GMP levels not only utilize the biofilm's physical barrier but also enhance bacterial stress resistance in both biofilm and planktonic states by upregulating *csgD* and stress-response genes, and by inducing low-metabolism survival strategies.

The relationship between c-di-GMP and virulence remains controversial [41]. This controversy stems from the generalized definition of virulence factors. Another reason for this controversy is the interspecies variation in the role of c-di-GMP. While some studies associate biofilms with increased virulence gene expression, others suggest that biofilm-embedded bacteria adopt a “stealth” phenotype to evade immune detection. Wang *et al.* demonstrated that bacterial pathogenicity is enhanced by phosphodiesterase-mediated degradation of c-di-GMP in *Salmonella* [42]. A subsequent study proposed that c-di-GMP competes with bacterial siderophores for binding to human lipocalin-2, thereby suppressing host antibacterial activity [43]. In contrast, other studies indicated that c-di-GMP binds to host immune proteins STING and DDX41, activating immune responses such as IFN-β and IP-10 release [44]. Our co-culture experiments demonstrated that Δ*dgcQ* (low c-di-GMP) enhanced *E. coli* adhesion to and invasion of HCT116 cells, while triggering robust proinflammatory responses (IL-1β, IFN-β, IP-10, and NF-κB) (Fig 7A–7G). Conversely, O*dgcQ* (high c-di-GMP) attenuated virulence factors, which in turn reduces immune system stimulation. Similar c-di-GMP-mediated modulation of host responses has been observed in the Gram-negative bacterium *Brucella* [45]. This dichotomy may reflect c-di-GMPʼs ability to modulate pathogen-associated molecular patterns (PAMPs): high c-di-GMP levels reduce flagellin and LPS exposure via EPS encapsulation and motility suppression, thereby limiting activation of immune receptors such as TLR5 and STING [46,47]. Emerging evidence suggests that virulence phenotypes predominantly drive acute infections by eliciting immune responses [48], while low-invasiveness bacteria evade immune recognition and clearance, facilitating chronic infections [45,49]. Therefore, we speculate that *E. coli* with high c-di-GMP levels may be more prone to immune escape by inhibiting host inflammatory responses, which is a strategy critical for persistent infections, particularly in biomaterial-associated contexts.

In summary, our study indicates that *dgcQ* serves as a central regulator of c-di-GMP levels in *E. coli*, shaping biofilm dynamics, stress resistance, and host-pathogen interactions. The results elucidate stage-specific roles of c-di-GMP in BAIs, suggesting that therapeutic strategies targeting this pathway require temporally precision. Early interventions could inhibit c-di-GMP degradation to suppress motility-driven colonization, while established biofilm require disruption c-di-GMP-mediated stress resistance or EPS synthesis to sensitize bacteria. The immunomodulatory effects of c-di-GMP further highlight its dual targeting potential—both in pathogens and host immune pathways. However, this study has limitations. First, the use of a single *E. coli* strain (ATCC25922) limits generalizability; future work should validate these findings in clinical isolates and other opportunistic pathogens. Second, focusing solely on PVC surfaces necessitates comparative studies across biomedical materials (e.g., silicone, polyurethane) to assess c-di-GMP's role in device-specific infections.

## 5 Future perspectives

Our work provides a foundation for developing anti-biofilm therapies tailored to disrupt this critical signaling pathway in BAIs. Targeting c-di-GMP signaling presents a promising strategy to mitigate BAIs. For instance, inhibiting DgcQ activity or promoting PDE expression could destabilize biofilms, rendering bacteria more susceptible to antibiotics and immune clearance. Conversely, engineered materials coated with c-di-GMP-modulating agents such as PDE mimics or DGC inhibitors might prevent bacterial adhesion or promote biofilm dispersal. Future *in vitro*, *in vivo*, and translational studies are required to validate the therapeutic potential of these findings against BAIs.

## Supporting information

S1 Table The sequence of primers for constructing and identifying *dgcQ* mutant strains. (DOCX)

S2 Table The sequence of primers for qRT-PCR. (DOCX)

S1 Fig Identification of ATCC25922 and ATCC25922Δ*dgcQ* strains. (DOCX)

S2 RAW images. (PDF)
