# Supplementary material for: The role of cyclic di-GMP in biomaterial-associated infections caused by commensal Escherichia coli
Source: PLoS One. 2025 Aug 20;20(8):e0330229. doi: 10.1371/journal.pone.0330229 (PMC12367115; doi:10.1371/journal.pone.0330229)
Supplement: S1 Table — (DOCX) [file pone.0330229.s001.docx]

**Table S1. The sequence of primers for constructing and identifying *dgcQ* mutant strains.**

| **Primers** | **Sequence (5′-3′)** |
| --- | --- |
| P1 | GTGCAGCACGAGACAAAAAT |
| P2 | AAGTGAACCAGCGTACCGACACACCATTAACTGGCA |
| P3 | CGGTACGCTGGTTCACTTCGCA |
| P4 | TTAAGCGTTATCGCTCGCGAATA |
| P5 | CGCGAGCGATAACGCTTAAGCTGTTTTGGCGGATGA |
| P6 | GTCGTCATCGTCTTTGTAGTCCATGGTTAATTCCTCCTGT |
| P7 | AGATTAGCGGATCCTACCTG |
| P8 | CACTTCTGAGTTCGGCATGG |
| P9 | GGCGTGAAGGGCTGGACCAT |
| gRNA1 | TCTGCCAGTTAATGGTGTGT |
| gRNA2 | ACGAGAAAATCGTCACCGCG |
