# Supplementary material for: The role of cyclic di-GMP in biomaterial-associated infections caused by commensal Escherichia coli
Source: PLoS One. 2025 Aug 20;20(8):e0330229. doi: 10.1371/journal.pone.0330229 (PMC12367115; doi:10.1371/journal.pone.0330229)
Supplement: S2 Table — (DOCX) [file pone.0330229.s002.docx]

**Table S2. The sequence of primers for qRT-PCR.**

| **gene** | **Forward (5′-3′)** | **Reverse (5′-3′)** |
| --- | --- | --- |
| *dgcQ* | AGTGGTTTTGCTGGAAGATG | AGAGGTGAAATCCAGTGGCG |
| *flhC* | ATGCTGCCATTCTCAACCGA | CAAACCGCACCAATGTCCAG |
| *flhD* | TTTTGACAGCCACCAGACGA | TCAGCAAGCGAGTTGAGAGC |
| *motA* | AGTTTGGTCGCAAAACGCTC | CCTCGGTTGTCGTCTGTTGT |
| *motB* | CCGAATTAGCCTCTCGGGTC | ACACTTTGCCGCCATTCAAC |
| *ycgR* | CAACATCGCCGTGCTAAAGG | ATGCCGGAAGCAGCAAGTAT |
| *csgA* | GTGGCGGTAACTCTGCTCTT | TCAGAACCTTGGCCCACATC |
| *csgD* | GCGGCGAATGCTACTTTACG | CCGATGAGTAAGGAGGGCTG |
| *bcsA* | GATGATCGCCCTGTTTGTGC | TCACCCAGTCGACGTACTCT |
| *ynfM* | AGCGACACTGACAAGCAAAG | AGAAGTGCGAAAGTTGCCAG |
| *sodA* | CCACCACACCAAACACCATC | GGTTTTCTTGTCTGCTGGCA |
| *katE* | GACGGTGATGTGAAAGGTCG | TCGGGAGTAGAGCAGTTTGG |
| *rstA* | TGGGTGCCTGCGACTATATT | CAGGGCTTTGTAGGGAGTCA |
| *ibpA* | TATCCCCGCTTTACCGTTCT | GCAATGCGGTAATGGTTTTCG |
| *ibpB* | GGCTGCATCAAGGGCTTATG | TGGGTTCAGGCTCATTACGA |
| *gadA* | GTCAAGTCCTGGACCTGTGA | CGATAGCTGGGGTTACGGTT |
| *gadB* | TTATTGCCGTGCTGCTGTTT | AATAGGGCGACAATAAGCGC |
| *hdeA* | GCATAAATTCGCCCGTTACT | ATGGTATTTTCGTCGCAGGC |
| *hdeD* | CAGACCTGGGACGACGATAA | CACAGATCGGCAACCATGTT |
| *16S rRNA* | CTTGCTGCTTTGCTGACGAG | GGTCCCCCTCTTTGGTCTTG |
