# Supplementary material for: The role of cyclic di-GMP in biomaterial-associated infections caused by commensal Escherichia coli
Source: PLoS One. 2025 Aug 20;20(8):e0330229. doi: 10.1371/journal.pone.0330229 (PMC12367115; doi:10.1371/journal.pone.0330229)
Supplement: S1 Fig — (DOCX) [file pone.0330229.s003.docx]

**Identification of strains ATCC25922 and ATCC25922Δ*dgcQ***

The *dgcQ* gene of *E. coli* ATCC25922 was amplified by PCR using primers P1 and P4. As shown in S1A Fig, the amplified fragment bands (lanes 1 and 2) were approximately 1700 bp. In contrast, the amplified fragment bands of the suspected ATCC25922Δ*dgcQ* (lanes 3 and 4) were about 1450 bp (S1B Fig ). The sequencing results were compared with the gene sequence provided on the ATCC official website, indicating that a gene fragment of 270 bp had been successfully knocked out. The expression of DgcQ in ATCC25922 and ATCC25922Δ*dgcQ* were detected by western blotting, shown in S1C Fig . the mean intracellular c-di-GMP in ATCC25922 and ATCC25922Δ*dgcQ* were detected by ELISA, shown in S1D Fig . All these results suggest that *dgcQ* gene is deleted and the function of DgcQ enzyme is lost.


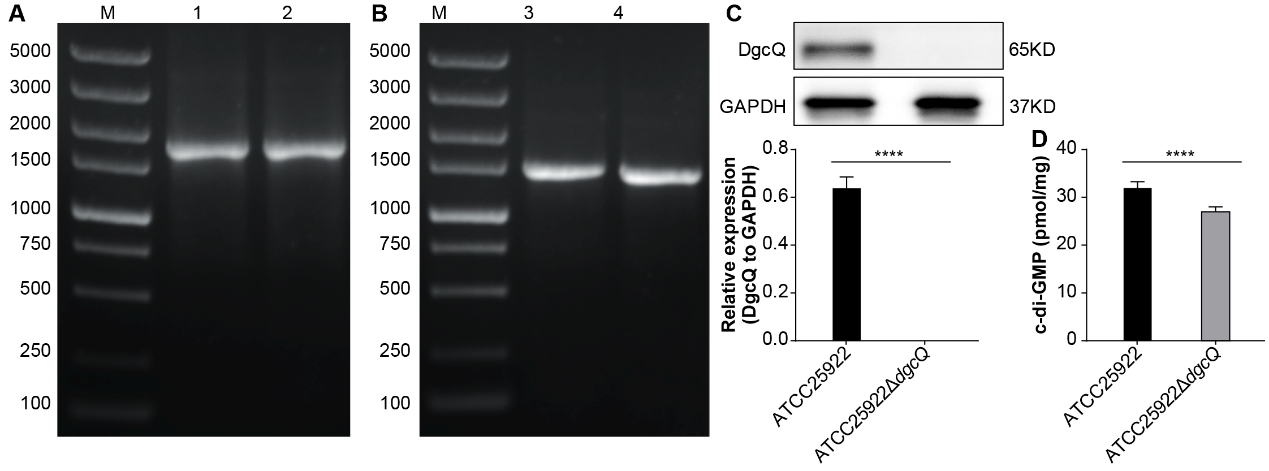


**Fig S1. Identification of the *E. coli* ATCC25922 strain and ATCC25922Δ*dgcQ* strain**. (A) PCR identification of the *E. coli* ATCC25922 strain. (B) PCR identification of the *E. coli* ATCC25922Δ*dgcQ* strain. Lane1, 2 amplified by primers P1, P4 (expected sizes: 1695 bp, 1695 bp). Lane3, 4 amplified by primers P1, P4 (expected sizes: 1425 bp, 1425 bp). M: 5000 bp ladder. (C) The DgcQ expression of ATCC25922 and ATCC25922Δ*dgcQ* strains (n=3). (D) The c-di-GMP levels of ATCC25922 and ATCC25922Δ*dgcQ* strains (n=3).
