# Supplementary figures and images for: The role of cyclic di-GMP in biomaterial-associated infections caused by commensal Escherichia coli
Source: PLoS One. 2025 Aug 20;20(8):e0330229. doi: 10.1371/journal.pone.0330229 (PMC12367115; doi:10.1371/journal.pone.0330229)

Figure 1A

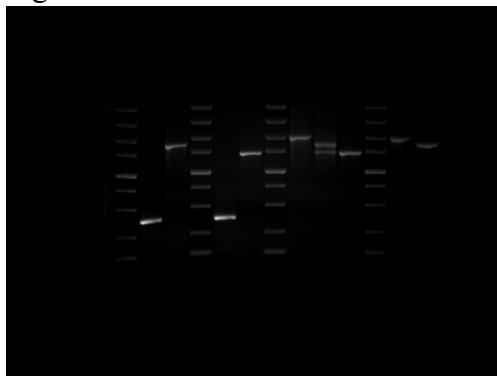

Figure 1D

DgcQ

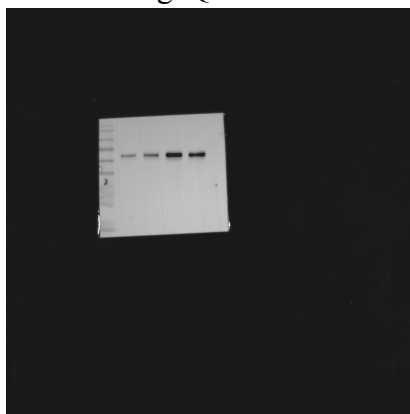

GAPDH

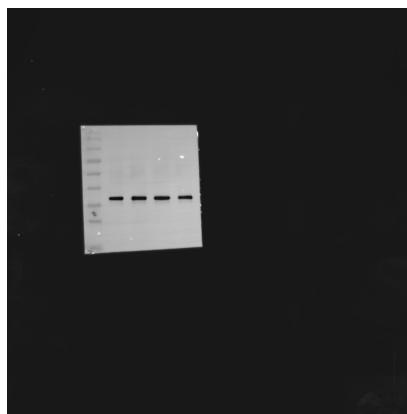

Figure 1E

DgcQ

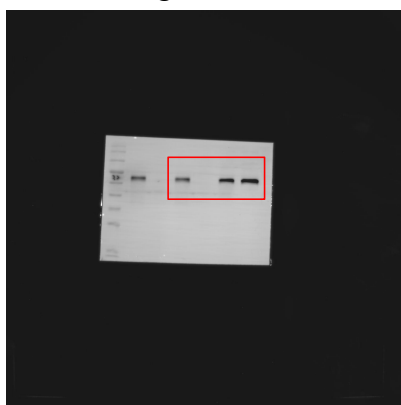

GAPDH

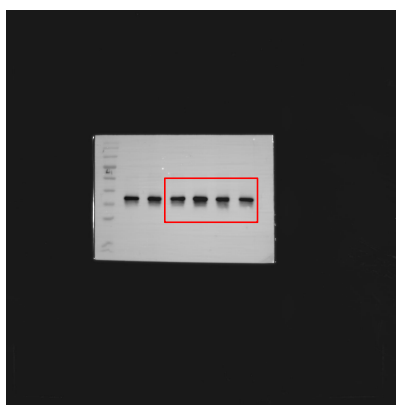

Figure S1A, 1B

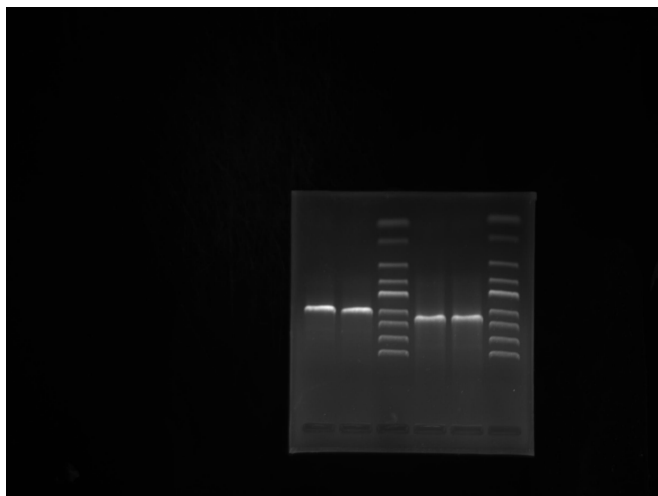

Figure S1C

DgcQ

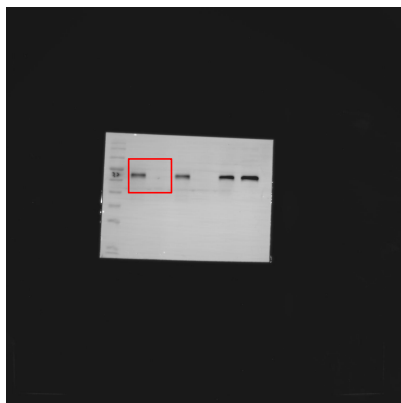

GAPDH

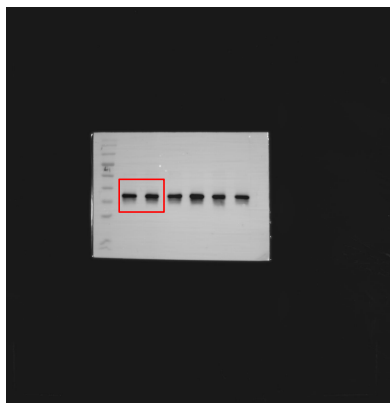

Supplement: S2 — (PDF) [file pone.0330229.s004.pdf]
